# Retraction: Gulf war illness inflammation reduction trial: A phase 2 randomized controlled trial of low-dose prednisone chronotherapy, effects on health-related quality of life

**DOI:** 10.1371/journal.pone.0322822

**Published:** 2025-04-15

**Authors:** The PLOS One Editors

After this article [1] was published, concerns were raised about the statistical methods used and the validity of the reported conclusions. Specifically, concerns were raised that between-group analyses would be needed to address the study aim, and that the within-group analyses reported in the article included only a subset of the data and did not align with the analysis method described in the IRB-approved study protocol. It was raised that when the full dataset is analyzed using between-group methods, there is no significant difference in HRQOL between placebo and prednisone treatment groups.

The clinical trial registration [2] describes the study as a double-blind, placebo-controlled trial for which outcome measures are defined by change from baseline, with data collection at 0, 8, and 16 weeks. The registry [2] also does not indicate a plan to do subgroup analyses, which are the focus of the article [1].

The corresponding author stated that the study was an IND exempt Phase 2 randomized clinical trial, and that within-group analyses are appropriate for a Phase 2 trial. They noted that results of between-group analyses were negative and so the authors changed to focus on analyzing intragroup changes from baseline to 8 weeks. The corresponding author also informed PLOS that they had identified a flaw in the statistical analysis of primary outcome data subgroups, and that reanalyses addressing this issue supported the article’s main conclusion about a statistically significant improvement in physical HRQOL in the treatment group.

PLOS received contradictory expert input on whether appropriate statistical methods were used in the reported study. A statistical reviewer advised that the test used in the article seems appropriate, and that subgroup analyses are acceptable provided they are reported along with the caveat that the study was not designed to detect subgroup differences. A member of the Editorial Board advised that the data should have been analyzed to compare the outcomes between the placebo and treatment groups using a paired sample t-test, and that when reanalyzed using this test there is no statistically significant difference between the placebo and treatment groups. The member of the Editorial Board also raised concerns about variables missing from the dataset provided with and analyzed in the article.

Although questions about the statistical analyses were not fully resolved in editorial follow-up discussions, PLOS concluded that the article’s main conclusions about a prednisone-associated improvement in physical HRQOL are not supported. The conclusions do not reflect the negative results of between-group (placebo vs. treatment) comparisons, and the reported results do not support that the improvement observed within the indicated prednisone treatment subgroup was statistically significant compared to changes observed in the placebo group.

Based on an independent investigation conducted at the Minneapolis Veterans Affairs Health Care System, the Department of Veterans Affairs found that the first author engaged in research misconduct (falsification of research) related to the submission of this article [1]. Specifically, in [1] the first author selectively used study results to suggest a benefit from prednisone, and omitted the results of the originally specified analysis which showed no significant difference between prednisone and placebo. Consequently, the results presented in [1] do not accurately represent the research record. As a result of the research misconduct findings, a request to retract the paper was submitted to *PLOS One*.

The *PLOS One* Editors therefore retract this article.

RRR agreed with the retraction. RRB did not agree with the retraction and stands by the article’s findings.
